# In silico characterization of the GH5-cellulase family from uncultured microorganisms: physicochemical and structural studies

**DOI:** 10.1186/s43141-021-00236-w

**Published:** 2021-09-30

**Authors:** Rahmat Eko Sanjaya, Kartika Dwi Asni Putri, Anita Kurniati, Ali Rohman, Ni Nyoman Tri Puspaningsih

**Affiliations:** 1 Mathematics and Natural Science Study Program, Faculty of Science and Technology, Kampus C Universitas Airlangga, Mulyorejo, Surabaya, East Java 60115 Indonesia; 2 University-CoE-Research Centre for Bio-Molecule Engineering, 2nd Floor ITD Building, Kampus C Universitas Airlangga, Mulyorejo, Surabaya, East Java 60115 Indonesia; 3 Chemistry Education Study Program, Faculty of Teacher Training and Education, Universitas Lambung Mangkurat, Jl. Brigjend. H. Hasan Basry, Banjarmasin, Kalimantan 70123 Indonesia; 4 Department of Health, Faculty of Vocational Studies, Kampus B Universitas Airlangga, Surabaya, East Java 60286 Indonesia; 5 Department of Chemistry, Faculty of Science and Technology, Kampus C Universitas Airlangga, Mulyorejo, Surabaya, East Java 60115 Indonesia

**Keywords:** Biofuel, Cellulase, Glycoside hydrolase 5 family, CelGH5, Uncultured microorganism, Computational tool, GenBank Database, Protein Data Bank

## Abstract

**Background:**

Hydrolysis of cellulose-based biomass by cellulases produce fermented sugar for making biofuels, such as bioethanol. Cellulases hydrolyze the β-1,4-glycosidic linkage of cellulose and can be obtained from cultured and uncultured microorganisms. Uncultured microorganisms are a source for exploring novel cellulase genes through the metagenomic approach. Metagenomics concerns the extraction, cloning, and analysis of the entire genetic complement of a habitat without cultivating microbes. The glycoside hydrolase 5 family (GH5) is a cellulase family, as the largest group of glycoside hydrolases. Numerous variants of GH5-cellulase family have been identified through the metagenomic approach, including CelGH5 in this study. University-CoE-Research Center for Biomolecule Engineering, Universitas Airlangga successfully isolated CelGH5 from waste decomposition of oil palm empty fruit bunches (OPEFB) soil by metagenomics approach. The properties and structural characteristics of GH5-cellulases from uncultured microorganisms can be studied using computational tools and software.

**Results:**

The GH5-cellulase family from uncultured microorganisms was characterized using standard computational-based tools. The amino acid sequences and 3D-protein structures were retrieved from the GenBank Database and Protein Data Bank. The physicochemical analysis revealed the sequence length was roughly 332–751 amino acids, with the molecular weight range around 37–83 kDa, dominantly negative charges with pI values below 7. Alanine was the most abundant amino acid making up the GH5-cellulase family and the percentage of hydrophobic amino acids was more than hydrophilic. Interestingly, ten endopeptidases with the highest average number of cleavage sites were found. Another uniqueness demonstrated that there was also a difference in stability between in silico and wet lab. The *II* values indicated CelGH5 and ACA61162.1 as unstable enzymes, while the wet lab showed they were stable at broad pH range. The program of SOPMA, PDBsum, ProSA, and SAVES provided the secondary and tertiary structure analysis. The predominant secondary structure was the random coil, and tertiary structure has fulfilled the structure quality of QMEAN4, ERRAT, Ramachandran plot, and Z score.

**Conclusion:**

This study can afford the new insights about the physicochemical and structural properties of the GH5-cellulase family from uncultured microorganisms. Furthermore, in silico analysis could be valuable in selecting a highly efficient cellulases for enhanced enzyme production.

## Background

Cellulases are a group of enzymes that have the ability to hydrolyze cellulose polymers into glucose monomers by hydrolyzing the β-(1 → 4) glycosidic bonds. Cellulases consist of three main enzymes: endo-β-1,4-glucanase (EC 3.2.1.4), β-glucosidase (EC 3.2.1.21), and exoglucanase. Exoglucanase consists of cellobiohydrolase I (EC 3.2.1.176) and cellobiohydrolase II (EC 3.2.1.91). Cellulases are classified into the carbohydrate acting enzymes (CAZy) in the group of the glycoside hydrolase (GH) [1]. Glycoside hydrolase (EC 3.2.1.-) is a well-known enzyme that hydrolyzes the glycosidic bond between two or more carbohydrates or between a carbohydrate and a non-carbohydrate moiety (http://www.cazy.org/Glycoside-Hydrolases.html). The grouping of enzymes into GH was based on conserved amino acid sequences and classified into several families [2–4]. Enzymes that are in the same family have similar amino acid sequences and three-dimensional structures. The GH5 family is the cellulase family, has at least 56 subfamilies, the largest glycoside hydrolase family [5]. Most of the GH5 members are multi-modular, including a catalytic module, substrate-binding module, and unidentified.

Cellulose is the most abundant biopolymer on Earth and is found in plant cell walls. It is a linear polysaccharide of glucose linked by β-1,4-glycosidic bonds. Cellulose is the main load-bearing polysaccharide, consisting of long chains of glucose strongly packed together due to H-bonds. It is embedded in a matrix of lignin, hemicelluloses, and pectin [6]. In addition to being highly abundant in plants, cellulose is also synthesized by some bacterial strains, such as *Acetobacter*, *Rhizobium*, *Xanthococcus*, *Pseudomonas*, *Azotobacter*, *Aerobacter*, and *Alcaligenes* [7]. Cellulose produced by bacterial strains is known as bacterial cellulose (BC). Animals (tunicates), algae, and protists can also produce cellulose [8]. As such, cellulose is the main target for renewable fuel production, such as bioethanol. The production of biofuel from renewable materials can provide economic and environmental benefits [9, 10]. However, bioethanol production using cellulosic materials requires high temperatures and harsh conditions [11, 12]. Hydrolysis of cellulosic materials and the saccharification process for bioethanol production enzymatically requires cellulase as it can perform under harsh conditions, such as high temperatures, high salinity, broad pH ranges, and stable in the presence of organic compounds [13–16].

Cellulases can be obtained from cultured and uncultured microorganisms. Cellulases from cultured microorganisms are defined as cellulases isolated by the cultivation of microorganisms under laboratory conditions. Cellulases produced from cultured microorganisms known as microbial cellulases [17, 18]. Cellulases were produced by microorganisms, such as *Aspergillus flavus* [19], *Bacillus* sp. [20], and other species of bacteria, fungi, and actinomycetes [16]. In contrast, the cultivation-independent (uncultured) technique is constrained by the fact that the majority of microorganisms, particularly those found in soil, cannot be cultivated in the laboratory [21]. Notably, much information is held within the genomes of uncultured microorganisms, and metagenomic technologies can investigate this potential [22]. Metagenomics is a method of analyzing and collecting functional genes from uncultured microorganisms or without the cultivation of microorganisms. It is an emerging approach to studying microbial communities in the environment [23]. Uncultured microorganisms represent a significant part of natural biodiversity. Microorganisms that can be cultured by standardized laboratory techniques comprise only 0.1–1% of the natural ecosystem [24–26]. Genes constructed based on metagenomic approaches have shown to be effective in identifying novel genes with specific activities [27–30].

The metagenomics-derived cellulases exhibit various characteristics and have commercial applications. Several members of the GH5-cellulase family have been identified using metagenomic approaches [28, 31, 32]. For example, a novel cellulase with unusual catalytic properties was isolated and characterized from a sugarcane soil metagenome (CelE1) [29] and CelGH5 from waste decomposition of oil palm empty fruit bunches (OPEFB) soil. CelE1 showed optimal activity at pH 7.0 and 50 °C with remarkable activity at alkaline conditions. Interestingly, CelE1 has a relative activity of 60% after incubation at 70 °C and has a higher activity at low temperatures (10–50 °C). This indicates that CelE1 is a thermotolerant enzyme with relative catalytic activity (> 65%) in the 10–70 °C temperature range. CelGH5 catalytic activity increased twofold after 4.0 M NaCl addition at pH 7.0, 55 °C. This indicates that CelGH5 is a halophilic with relative catalytic activity > 200% (unpublished data). Other cellulases from the metagenomic approach have unique properties, including cellulases from soil [27] and enriched culture from a hot spring [33], with hydrolytic activity increasing and stable in the presence of salt.

Understanding the properties and characteristics of cellulases can be achieved through their amino acid sequences and 3D structures. Therefore, predictions of cellulase properties can be considered an initial reference in developing the properties and characteristics of cellulases in the future. Most researchers’ current focus has been on the large-scale production of industrial enzymes for industrial purposes using multiple functional genes cloned on expression hosts. However, numerous variations—molecular weight, stability, amino acid composition, family, and secondary and tertiary structures—have been observed between different recombinant proteins produced from functional genes [34]. The availability of software and internet tools can be used to understand the overall physicochemical characteristics (i.e., primary, secondary, and tertiary structures, functional analysis, domains and motifs, and phylogenetic analysis) of the GH5-cellulase family from uncultured microorganisms. To date, no research has been conducted relating to the in silico analysis of the GH5 cellulase family from uncultured microorganisms. Only cellulases from *Bacillus* [34] and *Ruminococcus albus* [35] have been reported; these were analyzed using the in silico approach. Moreover, this information about the GH5 cellulase family from uncultured microorganisms retrieved from various tools and databases could be valuable in selecting a highly efficient strain for enhanced commercial enzyme production. The present study aimed to utilize in silico tools for the physicochemical and structural characterization of the GH5-cellulase family from uncultured microorganisms.

## Methods

### Sequence retrieval

Cellulase amino acid sequences from uncultured microorganisms were taken from GenBank, NCBI (http://www.ncbi.nlm.nih.gov/) based on the CAZy database belonging to the glycoside hydrolase family 5 on 2 September 2020. The sequences were kept in FASTA format, and unspecific or truncated sequences were removed. After reducing the data using the CD-HIT program (http://weizhong-lab.ucsd.edu/cdhit_suite/cgi-bin/index.cgi?cmd=cd-hit), 26 cellulases of GH5 family sequences were discovered. In addition, a sequence with an identity of CelGH5 was retrieved from University-CoE-Research Centre for Bio-Molecule Engineering (BioME), Universitas Airlangga, Surabaya, Indonesia, on 2 September 2020. CelGH5 sequence was obtained using the metagenomic approach from compost soil of palm oil waste and was also used in this study. Thus, a total of 27 different cellulase sequences were used in this study.

### Physicochemical properties

The physicochemical properties: molecular weight, theoretical pI, instability index, aliphatic index, and GRAVY were analyzed using ExPASy-ProtParam tools (https://web.expasy.org/protparam/) [36].

### Stability analysis

Stability analysis was done to CelGH5 for supporting the in silico data on the physicochemical properties. The pH and an additive stability assay for CelGH5 was carried out using the ThermoFluor assay. Protein melting temperature (*T*_*m*_) was determined by monitoring protein unfolding with the fluoroprobe, which emits fluorescence that can be quantified as a function of temperature when bound to hydrophobic protein domains [37]. The ThermoFluor assay was performed on a real-time PCR (RT-PCR) instrument (IT-IS Life Science Ltd., Ireland). Solutions of 2.5 μl of 80X SYPRO^TM^ Orange (Thermo Fisher, USA), 2.5 μl of 10 mg/ml CelGH5 enzyme, and 45 μl of test compound (buffer and additives) were added to the real-time PCR tube (GenFollower, China). Buffer test using a buffer screen of Britton-Robinson (BR) buffers [1:1:1 acetic acid:H_3_PO_4_:boric acid] ranging from pH 2.0–12.0 and protein buffer [50 mM phosphate pH 8.0, 250 mM NaCl, 5 mM Imidazole] as control. Additive test using 13 additives with water as control (Fig. 2). Samples were heated in real-time PCR from 37 °C to 97 °C in increments of 0.025 °C/s with initial and final holds were 10 s. The changes of the fluorescence were recorded every 0.025 °C using a fluorescence detector.

### Primary structure analysis

Amino acid composition, hydrophilic, and hydrophobic residues were calculated from the primary structure using the CLC main workbench 8.1.2 software (QIAGEN) [38]. The motifs or sequence consensus were identified using Multiple EM for Motif Elicitation (MEME) server (http://meme-suite.org/tools/meme) [39]. The maximum number of motifs was set as 6. It used a maximum width of 50 amino acids and a minimum width of 6 amino acids set along with other factors as default values.

### Secondary structure analysis

The secondary structure was obtained using Self-Optimized Prediction Method with Alignment (SOPMA) tool. The results obtained were the percentage composition of α-helix, β-sheet, turns, and random coil (https://npsa-prabi.ibcp.fr/cgi-bin/npsa_automat.pl?page=/NPSA/npsa_sopma.html) [40]. In order to confirm the predicted secondary structure, pictorial overviews of some experimental cellulase structures were retrieved from PDB RCSB, and the secondary structure was generated using PDBsum (http://www.ebi.ac.uk/thornton-srv/databases/cgi-bin/pdbsum/GetPage.pl?pdbcode=index.html). Additionally, information of its Ramachandran plots was generated by the PROCHECK tool on PDBsum.

### Tertiary structure analysis

The tertiary structures of four cellulases from uncultured microorganisms belonging to the GH5 family were determined. Structures with PDB ID 4EE9, 4HTY, 4M1R, and 5I2U were retrieved from PDB RCSB (https://www.rcsb.org/) on 2 September 2020, and their tertiary structures were further analyzed. QMEAN scores (https://swissmodel.expasy.org/qmean/) and ERRAT values (https://saves.mbi.ucla.edu/) were used to validate and evaluate the 3D structures. QMEAN4 was used to fit cumulative QMEAN values on a global scale at a range of 0 to 1 [41, 42]. ERRAT values were related to the resolution of protein structure. An average overall quality factor from ERRAT values around or higher 95% represents the high resolution of the structures, and the lower resolutions (2.5 to 3Ǻ) were approximately 91% [43, 44]. ProSA-web was used to assess the Z score and energy plots (https://prosa.services.came.sbg.ac.at/prosa.php). The desirable Z score should be < 1 compared to a nonredundant set of PDB structures [42, 45].

### Functional analysis

In order to determine the functional linkage and protein stability, the presence and absence of cysteine bonds (disulfide bonds) and their bonding pattern were predicted by CYS_REC (Softberry, Inc.) [46] (http://www.softberry.com/berry.phtml?topic=cys_rec&group=programs&subgroup=propt). The protein sequences of cellulase were analyzed by a conserved domain database (CDD) to determine conserved domains (https://www.ncbi.nlm.nih.gov/Structure/bwrpsb/bwrpsb.cgi) [47]. Potential cleavage sites were identified by using The Peptide Cutter tool (https://web.expasy.org/peptide_cutter/). The Peptide Cutter predicts potential cleavage sites cleaved by proteases or chemicals in a given protein sequence [48].

### Multiple sequence alignment and phylogenetic tree construction

The alignments of the amino acid sequences of cellulases were created using Clustal Omega (https://www.ebi.ac.uk/Tools/msa/clustalo/) [49–51] and generated by ESPript 3.0 program [52]. Cladograms of the GH5-cellulase family sequences from uncultured microorganisms were constructed through a maximum likelihood method based on the JJT matrix model [53] using the MEGA X software [54].

## Results

### Sequence retrieval

Twenty-six amino acid sequences were obtained from GenBank, and one sequence from our collection (Table 1) was added. Amino acid sequences were downloaded in FASTA format and used to analyze the physicochemical characteristics, primary and secondary structure, functional analyses, domains and motifs, and phylogenetic analyses.

**Table 1:** Details of selected sequences with their protein accessions

No.	Protein accessions	Name	Source	Length (aa)
1	ACA61132.1	Cellulase	Uncultured microorganism	553
2	ACA61135.1	Cellulase	Uncultured microorganism	552
3	ACA61137.1	Cellulase	Uncultured microorganism	546
4	ACA61140.1	Cellulase	Uncultured microorganism	537
5	ACA61144.1	Cellulase	Uncultured microorganism	512
6	ACA61145.1	Cellulase	Uncultured microorganism	532
7	ACA61149.1	Cellulase	Uncultured microorganism	520
8	ACA61152.1	Cellulase	Uncultured microorganism	346
9	ACA61160.1	Cellulase	Uncultured microorganism	518
10	ACA61162.1	Cellodextrinase	Uncultured microorganism	332
11	ACA61171.1	Cellobiosidase	Uncultured microorganism	386
12	ACH67609.1	Cellulase	Uncultured microorganism	345
13	ADB80100.1	Endoglucanase	Uncultured microorganism	532
14	ADB80110.1	Endoglucanase	Uncultured microorganism	343
15	ADB80112.1	Cellodextrinase	Uncultured microorganism	370
16	ADK55024.1	CelA	Uncultured microorganism	551
17	ADR64667.1	Cellulase	Uncultured microorganism	592
18	ADR64668.1	Cellulase	Uncultured microorganism	719
19	AEX97595.1	Cellulase	Uncultured microorganism	751
20	AEX97596.1	Cellulase	Uncultured microorganism	473
21	AFQ39736.1	Cellulase	Uncultured microorganism	559
22	AHB33631.1	Endo-1,4-β-D-glucanase	Uncultured microorganism	552
23	AHW46443.1	Cellulase	Uncultured microorganism	531
24	AOA60285.1	Cellulase	Uncultured microorganism	341
25	AOA60286.1	Cellulase	Uncultured microorganism	344
26	AOA60287.1	Cellulase	Uncultured microorganism	515
27	CelGH5 (this study)	Cellulase	Uncultured microorganism	333

### Physicochemical properties

Physicochemical properties of a protein, like molecular weight, pI, instability index, aliphatic index, and the average of hydrophobicity, are the preliminary properties to determine the uniqueness of proteins or enzymes [36]. The average molecular weight of GH5-cellulase family from uncultured microorganisms was 54862.07 Da or 54.86 kDa. The cellulase with the accession number of ACA61137.1 had a pI above 7 (pI > 7), 8.55, and another cellulase fell under 7 (pI < 7; Table 2). An isoelectric point (pI) below 7 (pI < 7) indicates the acidic nature of the protein. On the other hand, a pI of more than 7 depicts the alkaline nature. Negative charges (–R) of the sequences were computed based on numbers of aspartic acid and glutamic acid, while positive charges (+R) were based on numbers of arginine and lysine. Table 2 showed that the majority of cellulase had their pI lower than 7, indicating that the numbers of aspartic acid and glutamic acid for each cellulase sequence were more than arginine and lysine, except ACA61137.1 that had a pI > 7. Six sequences from the 27 selected sequences had *II* values of more than 40. This means that these sequences (ACA61162.1, ACA61171.1, ACH67609.1, AOA60285.1, AOA60286.1, and CelGH5) were predicted unstable in the test tubes. GRAVY index of cellulases had negative values ranging from −0.562 to −0.207. This result revealed that all GH5-cellulases from uncultured microorganisms had good interactions with water. The increasing positive scores indicated a greater hydrophobicity. The aliphatic index of a protein was defined using the aliphatic side chains such as alanine, valine, isoleucine, and leucine. It was a positive factor that could increase the thermostability of globular proteins [55]. The aliphatic index of the GH5-cellulase family was ranging from 62.20 to 84.28. The high aliphatic index refers to the fact that protein may be stable for a wide range of temperatures.

**Table 2:** Physicochemical properties computed using ExPASy-ProtParam tool

Accession	Mol. Wt. (Da)	pI	***II***	AI	GRAVY	−R	+R
ACA61132.1	62309.11	6.69	26.05	71.30	**−**0.538	66	65
ACA61135.1	61703.39	5.80	22.41	70.40	**−**0.497	66	60
ACA61137.1	60675.41	8.55	28.92	75.59	**−**0.415	56	60
ACA61140.1	60318.78	5.20	22.82	72.46	**−**0.467	70	53
ACA61144.1	56867.11	5.52	30.56	74.12	**−**0.353	61	51
ACA61145.1	58714.68	5.50	36.24	67.59	**−**0.451	60	49
ACA61149.1	57251.13	4.97	26.20	71.13	**−**0.312	67	48
ACA61152.1	39812.18	4.79	38.04	69.68	**−**0.273	46	29
ACA61160.1	56643.65	5.00	33.62	77.97	**−**0.233	68	49
ACA61162.1	38377.09	4.99	40.12	74.97	**−**0.401	53	36
ACA61171.1	45519.42	5.15	46.34	80.91	**−**0.549	60	43
ACH67609.1	39813.14	5.91	42.66	80.90	**−**0.344	44	36
ADB80100.1	59396.57	5.56	33.30	62.20	**−**0.498	60	49
ADB80110.1	39072.39	4.91	38.73	75.34	**−**0.239	46	29
ADB80112.1	43461.81	4.99	39.17	75.14	**−**0.562	60	38
ADK55024.1	62292.29	5.39	26.55	74.01	**−**0.501	71	60
ADR64667.1	64828.05	6.01	34.79	72.45	**−**0.329	74	65
ADR64668.1	79700.04	4.85	23.17	74.18	**−**0.372	91	61
AEX97595.1	83414.86	4.91	29.01	68.77	**−**0.480	91	65
AEX97596.1	51859.43	4.47	27.19	75.05	**−**0.207	56	29
AFQ39736.1	62735.57	6.30	23.73	72.61	**−**0.541	66	63
AHB33631.1	62552.79	5.60	26.59	75.85	**−**0.456	71	60
AHW46443.1	57361.16	5.16	26.56	66.57	**−**0.419	56	38
AOA60285.1	40643.19	5.59	44.78	84.28	**−**0.515	54	45
AOA60286.1	40450.78	5.25	44.09	82.21	**−**0.428	54	41
AOA60287.1	57844.96	4.68	33.97	82.37	**−**0.371	79	48
CelGH5 (this study)	37656.79	6.72	41.17	77.72	**−**0.303	39	38

### Stability analysis

**Fig. 1:**
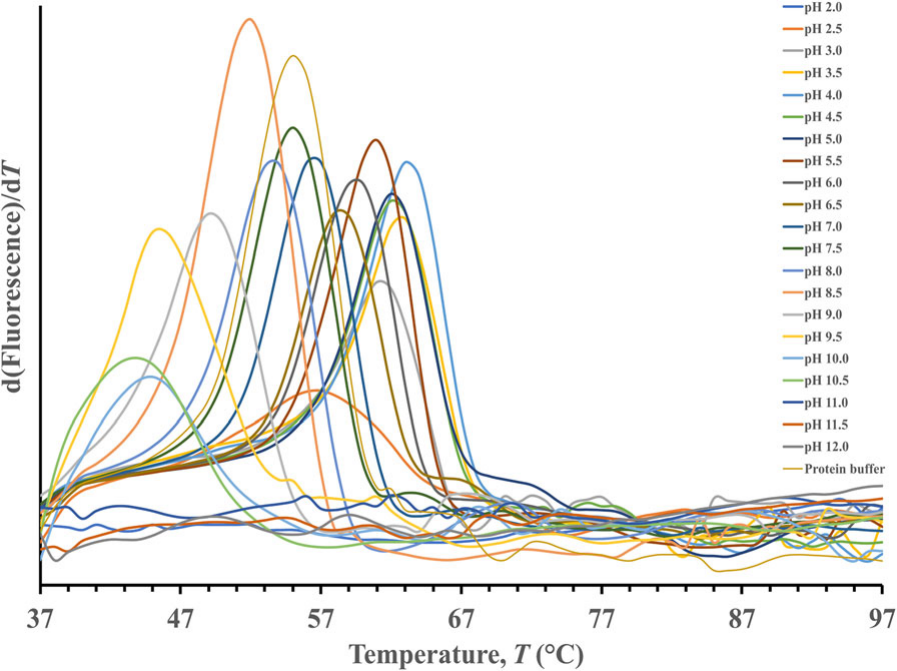
Thermostability analysis of CelGH5 in BR buffer at various pH values. The melting temperature (*T*_*m*_) is defined as the midpoint temperature of the protein folding–unfolding transition [56]. *T*_*m*_ is the first derivative of the fluorescence emission as a function of temperature (dF/dT). Here, *T*_*m*_ is represented as the highest part of the curve

**Fig. 2:**
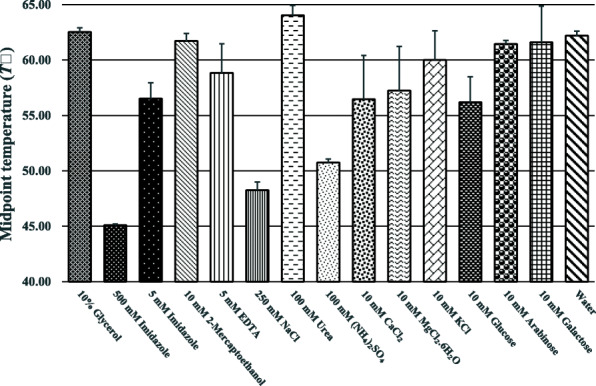
Midpoint temperatures of the protein-unfolding transition (*T*_*m*_) for CelGH5 in the presence of the additives. The control experiment is water, represented as a reference

CelGH5 gave *T*_*m*_ values at pH 2.5 to pH 11.0 and no apparent *T*_*m*_ values at pH 2.0, pH 11.5, and pH 12.0 (Fig. 1). These results indicate that CelGH5 has a wide pH range, from acidic to basic. At pH 4.0, the highest *T*_*m*_ value is obtained. This suggests that CelGH5 is more stable in acidic environments. Although it gives a *T*_*m*_ value in alkaline conditions (pH 7.5–11.0), the provided *T*_*m*_ is lower than the *T*_*m*_ of the control. Additives added to protein solutions could be stabilized or destabilized (Fig. 2). Thirteen additives were tested, and it was found that 7 additives gave a lower *T*_*m*_ value than the control or destabilizing properties. Imidazole, EDTA, NaCl, (NH_4_)_2_SO_4_, CaCl_2_, MgCl_2_.6H_2_O, and glucose were destabilized additives that should be avoided in CelGH5 storage. The other additives, glycerol, 2-mercaptoethanol, urea, KCl, arabinose, and galactose, had *T*_*m*_ values similar to the control.

### Primary structure analysis

**Fig. 3:**
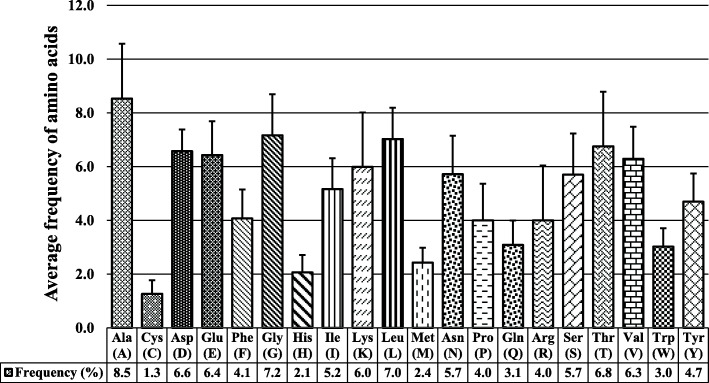
Amino acid composition of GH5-cellulases family from uncultured microorganisms computed using the CLC workbench 8.1.2 software

**Fig. 4:**
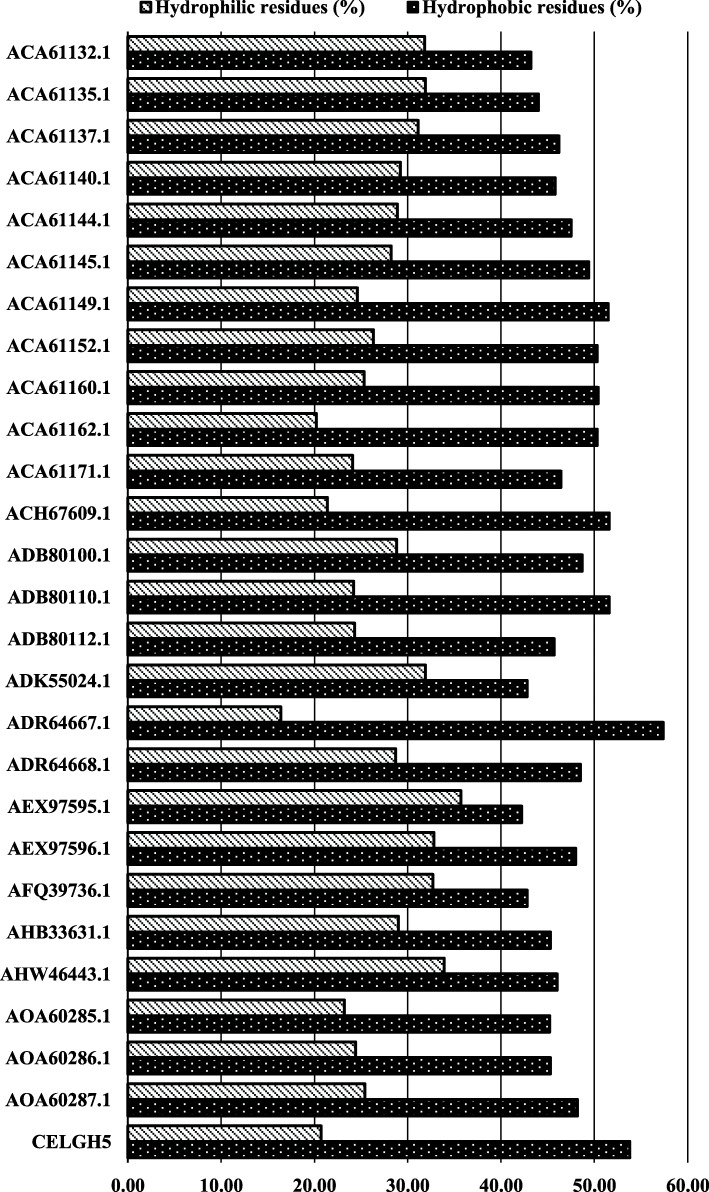
Frequencies of hydrophobic and hydrophilic residues. Hydrophobic residues: A, F, G, I, L, M, P, V, W; hydrophilic residues: C, N, Q, S, T, Y

Proteins differ from one another by their primary structures. Primary structure studies reveal the characteristics of all proteins. The amino acid composition of the GH5-cellulase family from uncultured microorganisms was determined using the CLC Main Workbench 8.1.2 software (QIAGEN). Figure 3 showed that alanine (8.5%) was the most abundant amino acid in all these sequences, followed by glycine (7.2%), leucine (7.0%), threonine (6.8%), aspartic acid (6.6%), glutamic acid (6.4%), and valine (6.3%). The composition of cysteine had the least quantity as compared to all amino acids. Figure 3 showed the comparative percentage average of amino acids in the GH5-cellulase family from uncultured microorganisms. Hydrophobicity was calculated by the number of hydrophobic residues (alanine, phenylalanine, glycine, isoleucine, leucine, methionine, proline, valine, tryptophan) and hydrophilic residues (cysteine, asparagine, glutamine, serine, threonine, tyrosine). All cellulase sequences analyzed were hydrophobic (Fig. 4). ADR64667.1 was a sequence with the highest hydrophobic residue percentage, whereas AEX97595.1 had the lowest.

MEME software can determine the conserved motif of a full-length protein. Table 3 showed six conserved motifs of all 27 sequences of the GH5-cellulase family from uncultured microorganisms. Five out of six motifs were identified as GH 5 family motifs, and there was no information for one motif.

**Table 3:** The six motifs of the GH5-cellulases family from uncultured microorganisms found among the 27 sequences

Length	Sequence	Occurrence at different site	Conserved domain
37	EMDTDGKVBDAWMARVKEVVDYVIDEGMYCIINVHHD	17	GH 5
41	TWRTTAQHETCWGQPVTKPELIKMMKEAGFGAIRVPVTWYQ	14	GH 5
33	YNTNKERYEKLWKQIAEEFKDYGQKLLFEAYNE	22	GH 5
41	YKAINSYAKSFVTTVRATGGNNATRNLIVNTYAASSTPNAM	17	GH 5
50	ALYAMDYLIKKAKEAGIGTFYWMGLSDGDYRSLPAFNQPDLAETJJKAYY	13	No information
50	HIIFQLHSYPNWQSESNAKSEIDNLISNIKSNLLNRAPVIIGEYATFTTW	7	GH 5

### Secondary structure analysis

The secondary structure contained α-helix, β-sheet or strand, and turns. However, one structure was not classified in the three usual groups; this was called a random coil. The SOPMA server analyzed the percentage or composition of α-helix, β-turn, extended strand, and random coils. Secondary structure analyses showed the percentage of each conformation. SOPMA revealed that the random coil was much greater than other secondary structures, such as helix, sheet, and turn. The random coil is usually described as a more flexible and dynamic folded chain region than other secondary conformational structures [57]. Table 4 showed the comparative percentage of α-helix, strands, β-turns, and random coil within all GH5-cellulase sequences. Sequences with accession numbers ACA61162.1, ACH67609.1, ADB80112.1, AOA60285.1, AOA60286.1, and CelGH5 had higher percentages of α-helix than random coils. The high alanine content might be due to the six sequences with more α-helix structures than other structures.

**Table 4:** Secondary structure among different sequences of GH5-cellulase family from uncultured microorganisms

Identity	Contents of principal secondary structure			
	α-helix (%)	Extended strand (%)	β-turn (%)	Random coil (%)
**Protein accession**^**a**^				
ACA61132.1	33.45	20.61	3.62	42.31
ACA61135.1	35.69	20.11	5.07	39.13
ACA61137.1	32.05	20.70	4.40	42.86
ACA61140.1	29.42	23.46	4.28	42.83
ACA61144.1	29.10	19.53	4.10	47.27
ACA61145.1	28.95	18.05	4.14	48.87
ACA61149.1	30.19	18.27	4.62	46.92
ACA61152.1	31.50	17.92	7.23	43.35
ACA61160.1	28.76	18.53	3.28	49.42
ACA61162.1	43.07	14.16	8.13	34.64
ACA61171.1	39.90	13.73	5.44	40.93
ACH67609.1	43.77	13.62	5.80	36.81
ADB80100.1	29.70	18.05	3.76	48.50
ADB80110.1	30.32	17.20	7.29	45.19
ADB80112.1	42.43	14.05	4.86	38.65
ADK55024.1	29.76	22.87	4.54	42.83
ADR64667.1	32.60	16.72	6.93	43.75
ADR64668.1	29.39	21.59	8.36	40.67
AEX97595.1	29.16	23.83	6.66	40.35
AEX97596.1	39.11	13.95	4.44	42.49
AFQ39736.1	30.41	21.47	5.01	43.11
AHB33631.1	27.72	20.11	4.53	47.64
AHW46443.1	31.83	16.95	3.58	47.65
AOA60285.1	42.23	15.25	6.74	35.78
AOA60286.1	43.02	14.24	6.40	36.34
AOA60287.1	27.57	20.39	8.93	43.11
CelGH5	42.34	14.71	5.71	37.24
Average frequency	33.83	18.15	5.48	42.54
**PDB ID**^**b**^				
4EE9	33.33	16.51	7.48	42.68
4M1R	36.49	18.58	8.78	36.15
5I2U	31.33	18.07	9.34	41.27
4HTY	25.91	15.60	7.52	50.97
Average frequency	31.77	17.19	8.28	42.77

^a^Predicted structure

^b^Experimental structure

**Fig. 5:**
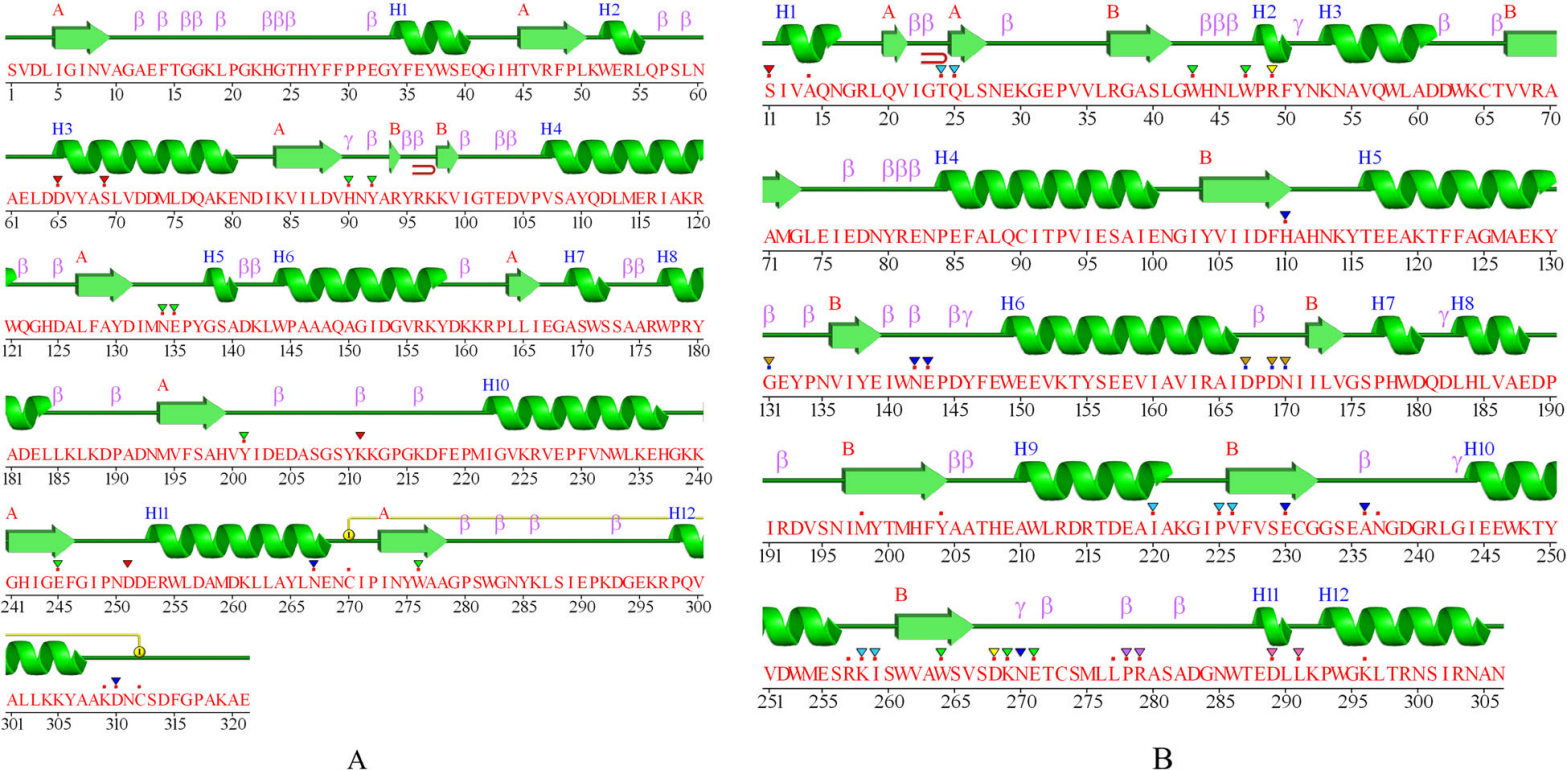
Schematic wiring diagrams of GH5-cellulase family from uncultured microorganisms. **a** PDB ID 4EE9. **b** PDB ID 5I2U. Helices structures labeled with H, strands, and β-turn labeled with A and β, respectively. The disulfide bridge is displayed as a yellow line. β-hairpin is labeled with a red hairpin

Cellulase structures with PDB ID 4EE9, 5I2U, 4M1R, and 4HTY were cellulases belonging to glycoside hydrolase family 5, recently identified via the metagenome approach. PDB ID 4EE9 was identified from the Antarctic soil [58], 5I2U was isolated from soil metagenome [27], 4M1R was from sugarcane soil [29], and 4HTY was from a metagenomic library. Commonly, cellulases from the GH5 family have a typical TIM-barrel fold consisting of α-helices and stranded parallel β-sheet as a core, and another secondary structure, like β-turn and coil. Table 4 showed the average conformational structures of cellulase dominated by random coils (42.77%), followed by α-helix (31.77%), strand (17.19%), and β-turn (8.28%). Figure 5 showed two schematic wiring diagrams of different cellulase structures. This figure confirmed from the predicted secondary structure that random coil had the highest content, followed by α-helix, strand, and β-turn. Disulfide bridges (Fig. 5a) connected cysteine residues 270 and 312. A small β-hairpin connected two strands in between residues 95 and 98. A small β-hairpin was also found in Fig. 5b that connected residues 22 and 25. There was no disulfide bridge found in Fig. 5b.

### Tertiary structure analysis

**Fig. 6:**
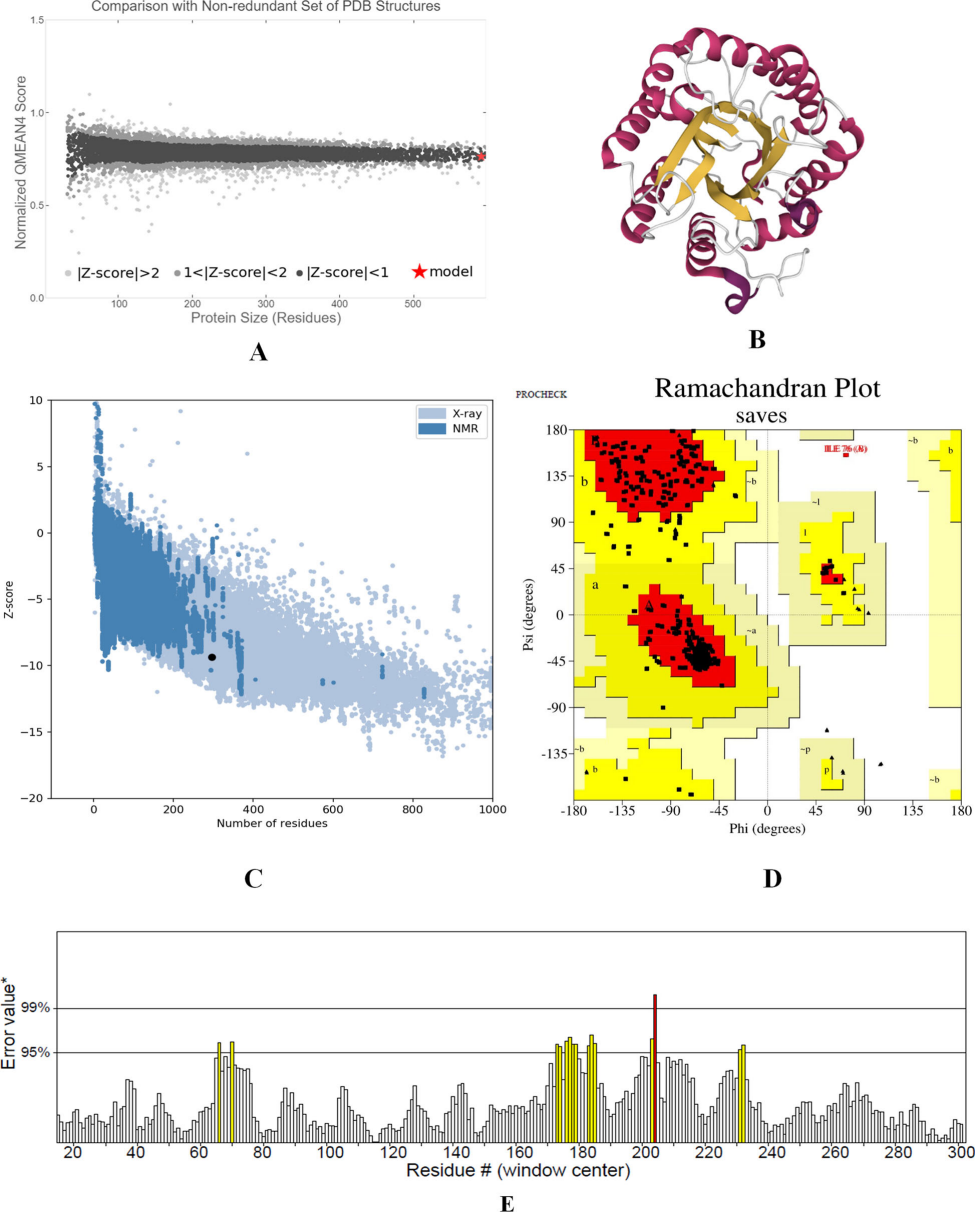
Visualization of cellulase tertiary structure (PDB ID 5I2U). **a** QMEAN4 Z score. **b** cellulase 3D structure. **c** Z score value generated by ProSA server. **d** Ramachandran plot showing the distribution of amino acids phi/psi angles. **e** ERRAT value showing structure resolution

The tertiary structures of the selected GH5-cellulase family were evaluated and assessed using computational tools. QMEAN4, ERRAT, Z score, and Ramachandran plot were quality parameters to assess and evaluate the tertiary structures of the GH5-cellulase family. There were four GH5-cellulase families from uncultured microorganisms that were structured (PDB ID 5I2U, 4EE9, 4M1R, 4HTY). Table 5 showed QMEAN4, ERRAT, Z score, and Ramachandran plot of four cellulase structures. A larger QMEAN4 score indicated a better structure, whereas negative scores referred to an unstable structure [45]. QMEAN4 predicted the global model structure quality based on a linear combination of four descriptors: local geometry, distance-dependent interaction, agreement of predicted secondary structure and solvent accessibility, and solvation potential. Figure 6a showed that the QMEAN4 scored 0.09, which represented a reliable 3D structure. The results also showed that the QMEAN4 Z score was compared to the nonredundant set of PDB structures. The QMEAN4 Z score of the structure was included in the group of PDB structures with a QMEAN Z score of less than 1.

**Table 5:** Comparison of QMEAN4, ERRAT, Ramachandran plot, and Z score for the quality assessment of three-dimensional structures

PDB ID	QMEAN 4 score	ERRAT quality factor (%)	Ramachandran plot				Z score
			Residues in favored region (%)	Residues in additional allowed region (%)	Residues in generously allowed region (%)	Residues in disallowed region (%)	
4EE9	0.54	96.154	89.7	10.3	0.0	0.0	−9.91
4M1R	0.40	95.606	88.1	11.9	0.0	0.0	−7.56
5I2U	0.09	94.965	87.7	11.9	0.0	0.4	−9.36
4HTY	0.11	96.530	90.0	9.7	0.3	0.0	−9.64

ProSA (Protein Structure Analysis) evaluated the accuracy of protein structure or model structure for prediction structure. The analysis was carried out based on statistical analyses of experimental protein structures, either by X-ray crystallography or NMR spectroscopy. The validation result of the 3D structure was a Z score. The 3D structure would be accurate if it had a Z score within the Z score range of the experimental protein structure [59]. Figure 6c showed that cellulase’s Z score was −9.36, which was included within the Z score range of the protein structure experimental with X-ray spectroscopy. ERRAT and Ramachandran plot were two other parameters to determine the quality of the tertiary structure. ERRAT values were related to structure resolutions. High resolution of 3D structures generally produces values around 95% or higher and lower resolutions would be present if the average overall quality factor is around 91%. Figure 6e reveals the overall quality factor of cellulase structure with the ERRAT value of 94.965%, a good enough structure resolution. A good quality model based on the Ramachandran plot would be expected to have over 90% in the most favored regions. The Ramachandran plot in Fig. 6d showed that residues in the favored region were less than 90%.

### Functional analysis

In this study, the cysteine residues were determined using the CYS_REC server. Table 6 reveals that among 27 protein sequences, 16 protein sequences contained cysteine residues connected by disulfide bonds. The presence of these disulfide bridges was regarded as a positive factor for stability at the molecular level. The amount of disulfide bonds was also calculated to determine the structure because of its role in protein folding. The CYS_REC server also determined the specific residue number connected by disulfide bonds between cysteine residues. For example, the sequence with accession number AEX97595.1 had more than one sequence of disulfide bridges.

**Table 6:** Disulfide bond prediction and conserved domain identification

Protein accession	Cys rec	Conserved domain	
		Domain	Position (aa)
ACA61132.1	Not SS-bounded	Cellulase	58-330
ACA61135.1	Not SS-bounded	Cellulase	58-326
ACA61137.1	Not SS-bounded	Cellulase	56-324
ACA61140.1	Not SS-bounded	Cellulase	56-324
ACA61144.1	61-477	Big_5	27-128
		Cellulase	187-480
ACA61145.1	22-174, 87-441, 325-473	BACON	36-120
		Cellulase	185-480
ACA61149.1	Not SS-bounded	Big_5	31-132
		Cellulase	185-492
ACA61152.1	33-313, 188-271	Cellulase	78-313
ACA61160.1	64-304	Big_5	32-130
		Cellulase	192-485
ACA61162.1	42-262	GH superfamily	55-311
ACA61171.1	21-255, 354-368	GH superfamily	65-357
ACH67609.1	Not SS-bounded	GH superfamily	25-320
ADB80100.1	161-325	BACON	34-117
		BACON	67-118
		Cellulase	185-480
ADB80110.1	26-206, 181-264	Cellulase	71-306
ADB80112.1	35-350	GH superfamily	57-353
ADK55024.1	11-62, 490-498	Cellulase	60-333
ADR64667.1	204-267, 241-502, 427-537	Cellulase	47-351
ADR64668.1	70-361	Cellulase	68-371
AEX97595.1	206-234, 238-333, 339-450, 562-731	Cellulase	64-347
		Dockerin_1	385-437
		CBM_4_9	478-569
		CBM_4_9	620-725
AEX97596.1	70-147, 262-344, 273-288	Cellulase	60-350
AFQ39736.1	Not SS-bounded	Cellulase	58-331
AHB33631.1	126-490	Cellulase	62-334
AHW46443.1	Not SS-bounded	Big_5	44-139
		Cellulase	186-480
AOA60285.1	77-81	Cellulase	31-315
AOA60286.1	Not SS-bounded	Cellulase	9-317
AOA60287.1	Not SS-bounded	Cellulase	91-333
CelGH5	Not SS-bounded	Cellulase	57-304

Table 6 showed the results of sequence analysis using CDD interactive web-based tools. It can be asserted that the sequence contained not only cellulase domains but also other domains. AEX97595.1 was the only cellulase with CBM among 27 sequences. AEX97595.1 had modular architecture, Cellulase - Dockerin_I - CBM_4_9. The presence of CBM could increase the binding capacity of cellulase to the substrate, indirectly helping the catalysis process of cellulose by cellulase. ACA61144.1, ACA61149.1, ACA61160.1, and AHW46443.1 had a Big 5 domain located before the cellulase domain. Meanwhile, ACA61145.1 and ADB80100.1 had the BACON domain. Another sequence had only cellulase domains without other domains. Information of conserved domains in cellulase sequence could be the engineering object to increase the ability or stability of cellulases.

**Fig. 7:**
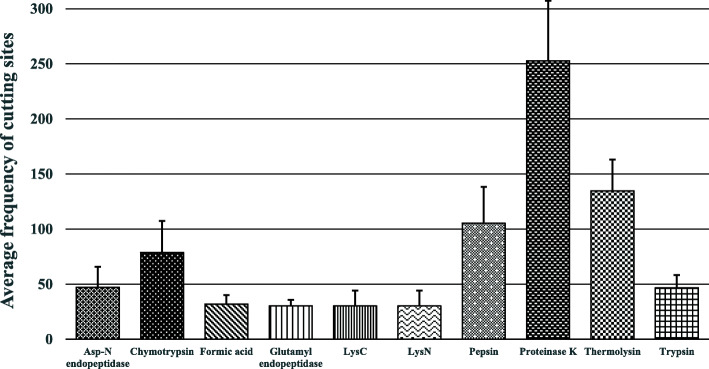
Average number of cleavage sites for the GH5-cellulase family from uncultured microorganisms as identified through the peptide cutter tool

Protease digestion is a valuable method for determining correct metabolism, enzymatic digestion, and high-order protein structure simplification. In addition to proteases, it is also important to identify chemicals that can cleave peptide chains. This study found teen endopeptidase/chemical that has the highest average number of cleavage sites in GH5-cellulase family sequences of the uncultured microorganisms. Those are Asp-N endopeptidase, chymotrypsin, formic acid, glutamyl endopeptidase, LysC, LysN, pepsin, proteinase K, thermolysin, and trypsin (Fig. 7).

### Multiple sequence alignment and phylogenetic tree construction

**Fig. 8:**
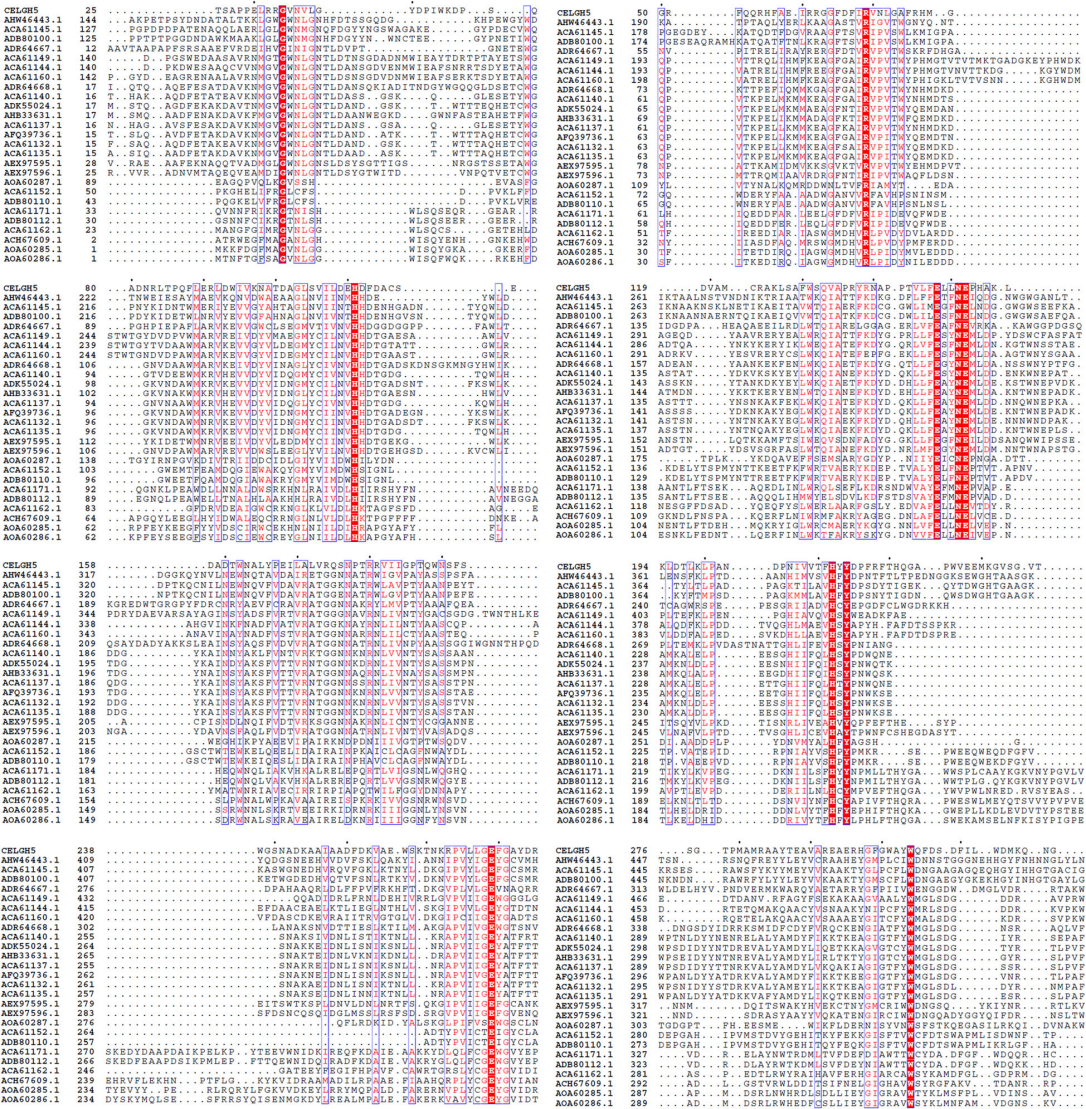
Multiple sequence alignment of GH5-cellulase family from uncultured microorganisms. Conserved residues in the red column

**Fig. 9:**
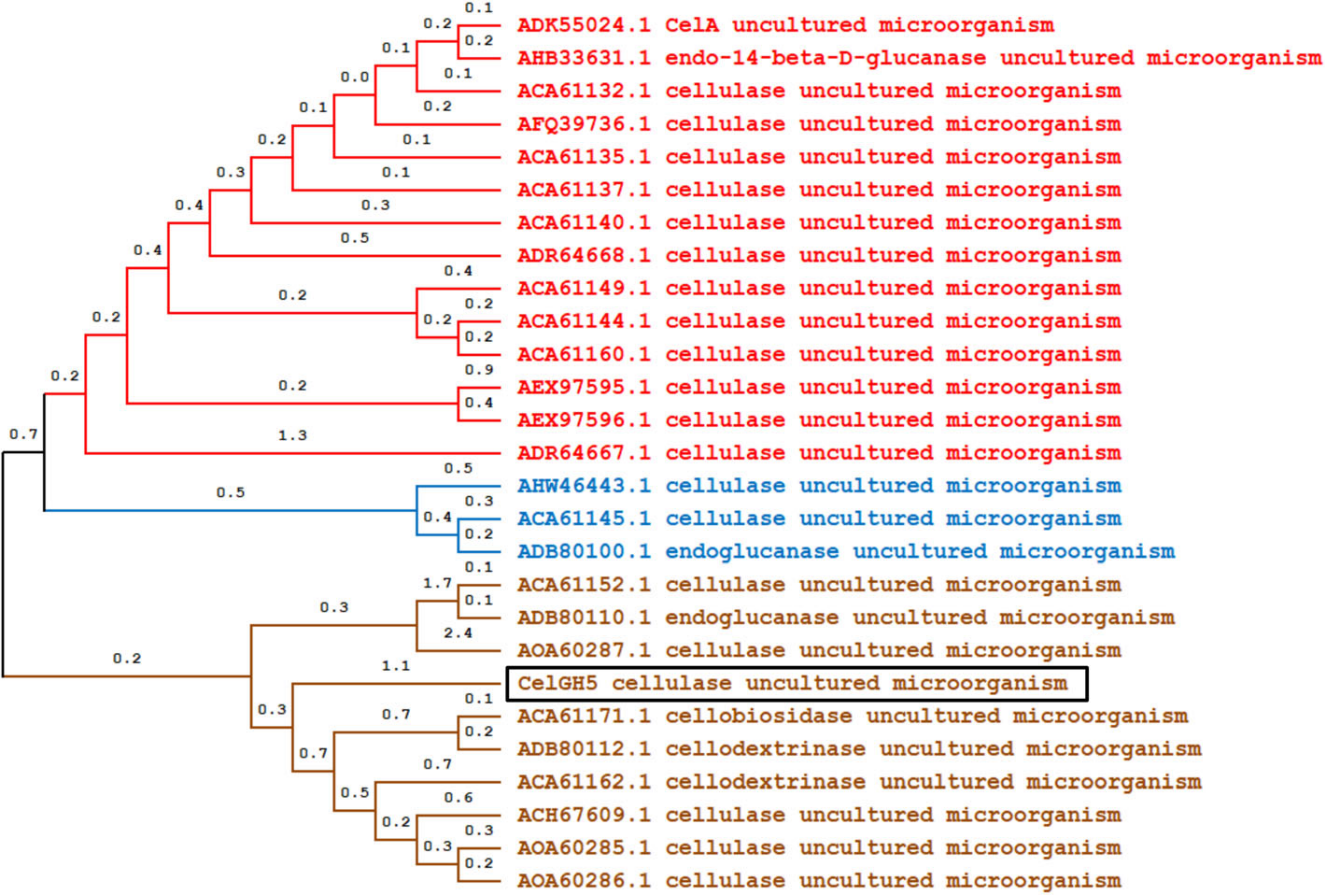
The cladogram of 27 different amino acid sequences of the GH5-cellulase family from uncultured microorganism by maximum likelihood method based on JTT matrix model using MEGA X with bootstrap replications are 1000 replicates

A multiple sequence alignment of retrieved cellulase sequences was performed by the Clustal Omega software and shown in Fig. 8. The sequence alignment identified several conserved amino acid residues (red column), like glycine (G), arginine (R), histidine (H), glutamic acid (E), asparagine (N), tyrosine (Y), and tryptophan (W). The most important residues in the GH5-cellulase sequence were two glutamic acids (E). The two glutamic acid residues had an important role in catalytic activity. Glutamic acid acted as a proton donor, and the other acted as a nucleophile [5, 60, 61]. Other residues had a role in stabilizing the structure and were also found in the cavity of active sites. Changes in amino acid residues in a conserved area could cause changes in the structure and function of these proteins. The phylogenetic tree of the GH5-cellulase family from uncultured microorganisms has been constructed with MEGA X using a maximum likelihood method based on the JTT matrix model with bootstrap replications are 1000 replicates. Figure 9 showed a cladogram of cellulase and distributed into three nodes. The dominant node consisted of 14 nodes and was marked in red lines. The second group consisted of 10 nodes and was represented by a brown line, including our sequence, CelGH5. The last group consisted of 3 nodes and was marked by blue lines.

## Discussion

An isoelectric point is a condition in which the protein surface is covered with no charge or the net charge, and thus the protein charge, is zero. At an isoelectric point, proteins or enzymes are compact and stable. The isoelectric point calculation is important for determining purification buffer systems focusing on an isoelectric and buffer systems for crystallization. The high efficiency and promising nature of protein crystallization can be improved by determining the pI of the protein, followed by screening for a buffer range at or near that pI value (within 2–3 pH units of the pI) [62]. In the current study, 27 cellulase sequences retrieved from GenBank had an isoelectric point (pI) values of less than 7, except the sequence with accession number ACA61137.1, which had a pI of 8.55. This result indicates that the GH5-cellulase family from uncultured microorganisms had acidic properties. Hoda et al. [35] found that GH5 cellulase from *Ruminococcus albus* had pI values ranging between 4.39 and 4.53, suggesting moderately acidic properties.

An analysis of halophilic/halotolerant enzymes revealed a consensus in which these enzymes tended to have more acidic or negative residues than their non-halophilic homologs [63]. The amount of glutamate and aspartate residues (**−**R) as acidic residues in the primary structure could not be used as references to determine the enzymes’ acidity or halophilic properties. The acidity or halophilic properties of enzymes could be determined from the glutamate and aspartate residues on the enzymes’ surfaces [27, 63, 64]; this would be known after determining the enzyme structure. The cellulase sequence analysis (PDB ID 5I2U) showed that 52 (16.7%) residues were acidic [27]. This result was relatively greater than that of other halophilic cellulases. The endoglucanase from *Bacillus subtilis* 168 (PDB ID: 3PZT) had 38 (11.6%) acidic residues [64], and the GH5 cellulases from *Thermoanaerobacterium*, which possessed halostable characteristics, only had 43 (11.3%) acidic residues [33]. CelGH5 possessed a slight difference between the negatively and positively charged residues. Despite this result, CelGH5 had halophile properties with a relative activity of more than 200% in the presence of 3M NaCl (data not shown).

The instability index (*II*) showed an estimation of the protein stability in a test tube. The instability index portrayed a stable protein when the index value was less than 40, and an unstable condition was shown when the index value was greater than 40. Six sequences from the 27 selected sequences had *II* values greater than 40. This means that these sequences (ACA61162.1, ACA61171.1, ACH67609.1, AOA60285.1, AOA60286.1, and CelGH5) were predicted unstable in test tubes. This result was in contrast with that of Duan et al. [65], whose paper showed that ACA61162.1 had an optimal condition at pH 4.5 and was stable a pH range of 3.5 to 10.5 based on experimental data [65]; in contrast, the *II* showed different results. The analysis of this condition shows that environmental aspects, such as the autoproteolysis of an enzyme, do not encapsulate the instability index calculation. Furthermore, the *II* model was only based on the primary sequence, and the secondary or tertiary structure contributions were not incorporated into the model [66]. Gamage et al. [66] calculated *II* values of three proteins; the results were consistent, similar to the degradation pattern observed by SDS-PAGE analyses. However, the unstable properties of α-S1-casein displayed in the *II* value were not related to the natural degradation visualized on SDS-PAGE analyses.

Based on the *II* value, CelGH5 was categorized as an unstable protein. Nevertheless, the ThemoFluor assay revealed that CelGH5 has a wide pH range of 2.5 to 11.0. In this pH range, CelGH5 gave an emission signal recorded by RT-PCR and converted to a melting point (*T*_*m*_). The increase in melting temperature under different buffers or additives gave rise to a thermal shift that quantified the stabilization of the protein [67]. At pH values of 2.0, 11.5, and 12, no apparent *T*_*m*_ was observed, indicating that the CelGH5 structure is destabilized at these pHs. CelGH5 had the highest *T*_*m*_ value at pH 4.0 or acidic conditions. Therefore, CelGH5 is suggested to be stored in a pH 4.0 buffer. Apart from the pH 4.0 buffer, an additive, such as glycerol, can be added to the CelGH5 solution because it does not affect the CelGH5 stability (Fig. 2). Glycerol is a cryoprotectant that helps stabilize proteins by preventing the formation of ice crystals at −20 °C, and thus the destruction of the protein structure. Other properties of CelGH5 include high stability with residual activity of 52% after 240 h incubation at 55 °C (data not shown). Thus, the results showed that the most important experimental condition is the careful use of the *II* to predict in vitro protein stability. This condition tells us that the *II* prediction does not accommodate all relevant information in the determination of protein stability under in vitro conditions. The application of *II* prediction toward protein stability still depends on the intrinsic nature of the protein and conditions of the protein milieu.

GRAVY analyses were calculated by adding the hydropathy values [68] of each amino acid residue and dividing by the length of full sequences. The GRAVY index represented the solubility of proteins and positive interactions with water [69]. The increasing positive scores indicated greater hydrophobicity. A low GRAVY value represented good interaction between water and protein. The GRAVY index of cellulases had negative values ranging from −0.562 to −0.207. This result revealed that all members of the GH5-cellulase family from uncultured microorganisms had good interactions with water. Although it was known that all analyzed cellulases had hydrophobic properties, it did not necessarily mean that they had a poor interaction with water. The GRAVY values and hydrophobic components of the amino acid sequence residues are a different matter. The hydrophobic residues in the formation of the three-dimensional structure are located inside or buried within the structure; thus, all surfaces interacting with water contain hydrophilic residues. Asparagine, cysteine, glutamine, serine, threonine, and tyrosine are hydrophilic amino acids that have a propensity to interact in the aqueous environment due to polarity properties; these residues are found on protein surfaces.

The high aliphatic index refers to protein stability under a wide range of temperatures. For example, the aliphatic index of the GH5 cellulase family ranged from 62.20 to 84.28. The higher the AI value, the greater the thermal stability of an enzyme. For example, the sequence with accession number AOA60285.1 was more stable than ADB80100.1. Interestingly, based on the *II* value, AOA60285.1 was an unstable enzyme. This result reinforces the notion that the use of the *II* as a reference in determining the stability of proteins or enzymes may also need to consider other influencing factors.

Primary structure analysis showed that alanine, glycine, leucine, threonine, aspartic acid, glutamic acid, and valine were the most abundant amino acids in the cellulase sequences analyzed. The number of cysteines was lower than other amino acids. Together with glycine, leucine, and glutamic acid, alanine had a greater tendency to build α-helix secondary structures in the protein conformation. This was in contrast with threonine and valine, which usually built β-sheet secondary structures. The aspartic acid had the role of connecting with the solvent, supported by hydrogen bonds. All analyzed cellulase sequences had hydrophobic properties because the majority of amino acid side chains had hydrophobic properties. Alanine, glycine, leucine, valine, proline, isoleucine, tryptophan, phenylalanine, and methionine had hydrophobic properties, and these amino acids were much more abundant than other amino acids.

MEME software revealed sequence motifs in all 27 sequences of the GH5-cellulase family, and a consensus of these sequences functioned as a signature sequence identifying the enzymes. Five out of six motifs were found, and one motif had no information. In order to confirm the conserved motif, an internet tool (https://www.genome.jp/tools/motif/) was used. With this tool, five motifs were confirmed as belonging to the GH5 family domain. The motifs also explained the diversity of the structures and functions of enzymes [70].

The SOPMA server analyzed the percentage of α-helix, β-turn, extended strand, and random-coil compositions. Secondary structure analyses displayed the percentage of each conformation. The coil structure had a higher percentage than other conformations. These results align with Hoda et al. [30], who found in cellulase from *Ruminococcus albus* that random coils were the most dominant secondary structure, followed by α-helix. The high percentage of coil might be caused by the high number of glycines and the presence of prolines [71]. A good glycine percentage in the sequence granted high flexibility to the polypeptide chain and provided structural rigidity. The properties of proline were created in a coiling structure because of the crinkly polypeptide chains that interfered with the secondary structures. Sequences with accession numbers, ACA61162.1, ACH67609.1, ADB80112.1, AOA60285.1, and AOA60286.1, had lower random coil percentages than α-helix structures. This was a result of the high number of alanines. These five sequences are likely to be present in cellulases from *Bacillus thuringiensis* and *Bacillus pumilus* [34], which have a higher α-helix structure percentage than other secondary structures.

The different amino acid sequences influenced the properties and formed different structures. Alanine, glutamic acid, and leucine were uncharged amino acids that played a significant role in the high helix-forming propensities. In contrast, glycine and proline had only a few helix-forming propensities [72]. Proline did not have any amide hydrogens; thus, it could not donate any amide hydrogens. However, it could break or bend the helix structure; additionally, the side chains could be disrupted because of the steric position of the backbone of the preceding turn inside a helix [73]. Proline was also found in the edge strands of β-sheets and existed presumably to avoid an “edge-to-edge” protein association that might have led to aggregation and amyloid formation. Proline was seen as the first residue of the helix due to the rigidity of the structure. However, glycine also disturbed the flexibility conformation of α-helical structures. Tyrosine, phenylalanine, tryptophan (a large aromatic group residue), threonine, valine, and isoleucine (β-branched amino acids) were mostly found in the middle of β-sheets [74].

The secondary structure β-turns had the lowest percentage. β-turns or reverse turns usually connected different antiparallel β-strands. The β-turn was stabilized by hydrogen bonds connecting the carbonyl oxygen and amide hydrogen. The β-turn was arranged into the four amino acids with the carbonyl oxygen as the first residue and the amide hydrogen as the fourth residue. Glycine and proline tended to have arrangements of β-turns. Proline had a crucial role in building the cis conformation that supported the β-turn formation. Contrastingly, glycine just had a small R group that allowed for high flexibility. There are some theories concerning the role of β-turns in globular proteins. First of all, β-turns had weak bonds that could not support the secondary structures. Second, β-turns played a role in the folding process. However, both of these perspectives were still inaccurate and required further supporting experiments.

There were four GH5 cellulases from uncultured microorganisms that had been structured (i.e., PDB ID 5I2U, 4EE9, 4M1R, 4HTY). Cellulases from the GH5 family had a typical TIM-barrel fold consisting of α-helices and β-sheets as a core structure, combined with other secondary structures, such as a β-turns and coils. Figure 6b displays the tertiary structure of cellulases obtained using the metagenome approach (PDB ID 5I2U), with halophile properties. The evaluation and quality assessment of structures were performed with the QMEAN4, ERRAT, Z score, and Ramachandran plot. The QMEAN score revealed geometric aspects of the protein structures and the global arrangement of variable residues. A larger QMEAN4 score indicated a better structure, whereas negative scores referred to an unstable structure [45]. QMEAN4 predicted the global quality of model structure based on a linear combination of four descriptors: local geometry, distance-dependent interaction, agreement of predicted secondary structure and solvent accessibility, and solvation potential. The QMEAN4 of cellulase’s 3D structures are represented in Fig. 6a. They depicted that the proteins were properly folded into a compact three-dimensional field. QMEAN4 scores of all cellulase structures varied from 0.09 to 0.54 (Table 5). Desirable QMEAN scores were 0–1 [42, 75]. The results also show that the QMEAN4 Z score was compared to the nonredundant set of PDB structures. The QMEAN4 Z score of the structure was included in the group of PDB structures, with a QMEAN Z score of less than 1. The verifications of the 3D structures were determined through crystallography represented by ERRAT values. ERRAT values were related to structure resolutions. ERRAT was also useful for analyzing protein structures from the numbers of non-bounded residues with a cutoff of 3.5 Å between different pairs of atoms. The high 3D structure resolution generally produces values of approximately 95% or higher. Lower resolutions would be present if the average overall quality factor were roughly 91%. Figure 6e displays the overall quality factor of the cellulase structure, with an ERRAT value of 94.96%, a good enough structural resolution. ERRAT values under 91% indicated that the structure had a lower resolution of approximately 2.5 to 3.0 Å. The Ramachandran plot was constructed to show the positions of each amino acid residue (Fig. 6d). Analysis of the Ramachandran plot (PDB ID 5I2U) showed that 87.7% of residues were present in the most favored region (Table 5). Residues in the favored region of the Ramachandran plot equaling more than 90% represented a good quality structure [76, 77].

The cysteine was an amino acid that played an important role in determining the thermostability of proteins. Cysteine-cysteine residues, creating a disulfide bridge, could influence the stability and folding of proteins. This was caused by an oxidative folding process occurring in the thiol groups of cysteine. Some studies showed strategies to increase protein stability by mutating cysteine. When the native disulfide bond was removed, the stability decreased. Besides, adding disulfide bonds also improved the rigidity and stability of protein structure [78]. The presence of disulfide bridges was regarded as a positive factor for stability at the molecular level [79]. The successful disulfide-bonding analysis supported the accuracy of 3D enzyme structure prediction [80]. The cleavage of disulfide bonds affected the native conformation and biological function. Thus, failed folding of the formation caused by disulfide bonds may have been due to protein aggregates [81].

The peptide cutter tool found 27 proteases and chemicals that can cleave GH5-cellulase sequences from uncultured microorganisms. From the 27 proteases and chemicals, there are 10 that possess the highest average number of cleavage sites, including Asp-N endopeptidase, chymotrypsin, formic acid, glutamyl endopeptidase, LysC, LysN, pepsin, proteinase K, thermolysin, and trypsin. Meanwhile, caspase 1, caspase 2, caspase 4, caspase 6, and enterokinase are proteases with the lowest cleaving ability. The results of the peptide cutter tool cleavage sites could be useful when conducting studies on a portion of a protein, separating domains in a protein, and removing a tagged protein while expressing a fusion protein [57].

The conserved domain position had an important role in determining the catalytic site of the observed sequences. Through this process, other functional domains in the sequence could be identified. CDD is a protein database that lists all proteins that have been registered or deposited using multiple sequence alignment models and full-length proteins. This database can also be used for the fast identification of proteins by looking at conserved domains in the protein sequence and classifying them into their respective families [47]. Based on the results, it was found that the sequence did contain not only cellulase domains but also other domains. The selected amino acid sequences had Big 5, BACON, Dockerin, and the carbohydrate-binding module (CBM). The presence of CBM could increase the binding capacity of cellulases to the cellulase substrate, indirectly helping the catalysis process [82]. The BACON domain was found in varied domain architectures and associated with various domains, including proteases and carbohydrate-active enzymes. The function of the BACON domain had an unclear relationship with carbohydrate metabolism but a strong connection to protease domains [83]. Dockerin is a domain that belongs to the cellulosome complex. Cellulosomes are multienzyme complexes with cellulosic activity and are usually found in anaerobic bacteria [84–87]. The sequence with accession number AEX97595.1 had a dockerin domain and was predicted as a bacterial cellulase-typical sequence. The bacterial immunoglobulin-like (Big) domain can be widely found in bacterial proteins with diverse biological functions such as adhesion and biofilm development [88].

Glycine, arginine, histidine, glutamic acid, asparagine, tyrosine, and tryptophan were conserved residues identified by the Clustal Omega software. These conserved residues played a pivotal role in the catalytic mechanism and were reported as cellulases from uncultured microorganisms or metagenomic approaches [28, 33, 58, 89]. Glutamic acid played an essential role in the GH5 family as a catalytic residue. Glutamic acid acted as a base or a catalytic nucleophile and a catalytic proton donor [90]. Three glutamic acid residues were found from multiple sequence alignments as conserved residues. Residues E148, E152, E269 were conserved glutamate from the CelGH5 sequence. It was predicted that E148 was the CelGH5 catalytic residue that acted as a proton donor, with E269 acting as a nucleophile. This prediction could be confirmed after determining the CelGH5 structure or aligning its sequence with other sequences whose structures had been determined. Histidine, asparagine, and tyrosine were conserved residues located between two catalytic residues. It was assumed that these residues were located in the CelGH5 cavity site that participated in substrate binding, stability, and hydrogen bond formation between catalytic residues and substrates [33, 58]. Histidine and tyrosine were conserved residues in the catalytic cavity site of cellulases from the soil metagenome library from Antarctica [58]. Glycine, arginine, and tryptophan played a role in the binding of the substrate and influenced hydrolysis activities [91].

The phylogenetic tree of GH5-cellulase was distributed into three nodes, with the dominant node consisting of 14 nodes and the minor nodes consisting of 3 nodes (Fig. 9). Every branch represented evolutionary lineages changing over time, and each lineage had a unique history [44]. CelGH5 clustered in the second group formed a new root and was a direct branch approaching the point of its ancestor. This indicates that CelGH5 is a metagenome GH5-cellulase sequence with a different typical sequence compared to other GH5-cellulase metagenome sequences. The cladogram branches further diverged into small branches, with every branch representing an evolution by the cellulases and each lineage having a unique history [44]. The vertical lines connecting horizontal lines revealed their irrelevance. The GH5-cellulase sequences from uncultured microorganisms diverged into three main daughter lineages; small branches resulted from the daughter branches. Branch length represented genetic changes among the sequences.

## Conclusions

The present study provided new insight on in silico study to determine the characteristics of cellulases from uncultured microorganisms belonging to the GH5 family of the CAZy classification in terms of their physicochemical and structural properties. The sequence length was roughly 332–751 amino acids and had a molecular weight range around 37–83 kDa. Based on the amino acid charge, the dominant-selected cellulase sequences had negative charges and pI values below 7 (acidic). Alanine was the most abundant amino acid making up the GH5-cellulase family, and the percentage of hydrophobic amino acids was more than hydrophilic. Interestingly, ten endopeptidases with the highest average number of cleavage sites were found. Another uniqueness demonstrated that there was also a difference in stability between in silico and wet lab. The *II* values indicated CelGH5 and ACA61162.1 as unstable enzymes, while the wet lab showed they were stable at broad pH range. The predominant secondary structure was the random coil, with an average percentage of 42.54%. The tertiary structure of four cellulase structures from the metagenomic GH5 family has fulfilled the 3D-protein structure quality based on QMEAN4, ERRAT, Z score, and residues in the favored region on the Ramachandran plot. Glycine, arginine, histidine, glutamic acid, asparagine, tyrosine, and tryptophan were conserved residues found from multiple sequence alignments. This study is significant as a consideration in terms of further isolation, characterization, and selection of a highly efficient cellulases for enhancing enzyme production.

## Data Availability

The authors declare that all generated and analyzed data have been included in the article.

## Abbreviations

GH5: Glycoside hydrolase family 5
CAZy: Carbohydrate-Active Enzymes database
OPEFB: Oil palm empty fruit bunches
GRAVY: Grand average of hydropathicity
pI: Isoelectric point
*II*: Instability index
AI: Aliphatic index
−R: Number of negative residues (aspartic acid and glutamic acid)
+R: Number of positive residues (arginine and lysine)
aa: Amino acid
MEME: Multiple EM for motif elicitation
SOPMA: Self-Optimized Prediction Method with Alignment
CDD: Conserved Domain Database
CBM: Carbohydrate-binding module
BACON: Bacteroidetes-associated Carbohydrate-binding Often N-terminal
Big: Bacterial immunoglobulin-like domain
QMEAN: Qualitative model energy analysis
MEGA: Molecular Evolutionary Genetics Analysis
NCBI: National Center for Biotechnology Information
PDB: Protein Data Bank
RCSB: Research Collaboratory for Structural Bioinformatics
